# A dynamic multitask evolutionary algorithm for high-dimensional feature selection based on multi-indicator task construction and elite competition learning

**DOI:** 10.3389/frai.2025.1667167

**Published:** 2025-10-20

**Authors:** Jinxin Tie, Chunfang Yan, Maosong Li, Jianqiang Gong, Yujie Wu, Hailin Fang, Meng Li, Weiwei Zhang, Jie Li

**Affiliations:** ^1^Ningbo Cigarette Factory, China Tobacco Zhejiang Industrial Co., Ltd., Ningbo, China; ^2^Technology Center, China Tobacco Zhejiang Industrial Co., Ltd., Hangzhou, China; ^3^College of Tobacco Science and Engineering, Zhengzhou University of Light Industry, Zhengzhou, China; ^4^College of Computer Science and Technology, Zhengzhou University of Light Industry, Zhengzhou, China

**Keywords:** feature selection, evolutionary multitask optimization, elite competition, knowledge transfer, high-dimensional data, tobacco data analytics

## Abstract

High-dimensional data often contain noisy and redundant features, posing challenges for accurate and efficient feature selection. To address this, a dynamic multitask learning framework is proposed, which integrates competitive learning and knowledge transfer within an evolutionary optimization setting. The framework begins by generating two complementary tasks through a multi-criteria strategy that combines multiple feature relevance indicators, ensuring both global comprehensiveness and local focus. These tasks are optimized in parallel using a competitive particle swarm optimization algorithm enhanced with hierarchical elite learning, where each particle learns from both winners and elite individuals to avoid premature convergence. To further improve optimization efficiency and diversity, a probabilistic elite-based knowledge transfer mechanism is introduced, allowing particles to selectively learn from elite solutions across tasks. Experimental results on 13 high-dimensional benchmark datasets demonstrate that the proposed algorithm achieves superior classification accuracy with fewer selected features compared to several state-of-the-art methods. Across 13 benchmarks, the proposed method achieves the highest accuracy on 11 out of 13 datasets and the fewest features on eight out of 13, with an average accuracy of 87.24% and an average dimensionality reduction of 96.2% (median 200 selected features), clearly validating its effectiveness in balancing exploration, exploitation, and knowledge sharing for robust feature selection.

## 1 Introduction

Feature selection has long been recognized as a critical step in machine learning and data mining, particularly when dealing with high-dimensional datasets. By identifying the most informative and non-redundant subset of features, feature selection not only improves model performance and interpretability but also significantly reduces computational costs. However, in high-dimensional spaces, the feature selection process becomes increasingly challenging due to the curse of dimensionality, feature redundancy, and complex interactions among variables.

Existing feature selection methods are broadly categorized into filter-based and wrapper-based approaches based on whether they rely on classifiers for subset evaluation. Filter methods assess features independently of any learning model, offering high efficiency and scalability, making them suitable for large-scale datasets (Kamalov et al., 2025). Despite these advantages, their inability to consider interactions with learning algorithms often limits their performance in downstream classification tasks. In contrast, wrapper methods evaluate feature subsets by training predictive models, leading to better classification accuracy but incurring high computational costs, especially in high-dimensional settings (Sadeghian et al., 2025).

Swarm intelligence algorithms, such as particle swarm optimization (PSO) and competitive swarm optimizer (CSO), have demonstrated strong potential in handling complex feature selection tasks (Huda and Banka, 2019; Tran et al., 2019; Swesi and Bakar, 2019; Song et al., 2021; Too et al., 2019). These methods mimic social behaviors to search for optimal feature subsets by balancing exploration and exploitation. However, standard PSO- and CSO-based algorithms often face issues such as slow convergence or premature stagnation when applied to datasets with thousands of features (Ding et al., 2020; Tran et al., 2018; Pichai et al., 2020; Li et al., 2023). To address these challenges, recent studies have turned to Evolutionary Multitasking (EMT), which leverages the latent synergy among multiple tasks to accelerate search efficiency and improve generalization performance (Chen et al., 2020; Chen et al., 2021; Li et al., 2023). However, most existing EMT-based feature selection methods still rely on fixed task definitions and lack adaptive mechanisms to dynamically construct tasks, evaluate task relevance, and selectively transfer knowledge. As a result, they are prone to negative transfer and limited scalability when applied to ultra-high-dimensional problems. Moreover, they rarely incorporate explicit competition mechanisms to maintain population diversity, which increases the risk of premature convergence.

To address these challenges, a novel dual-task multitask learning with competitive elites (DMLC-MTO) framework is proposed for high-dimensional feature selection. The core idea is to co-optimize a global task that retains the full feature space and an auxiliary task that operates on a reduced subset of features generated by multi-indicator integration. The optimization is driven by a competitive particle swarm mechanism with hierarchical elite learning and inter-task knowledge transfer. This approach aims to balance global exploration and local exploitation while leveraging the shared knowledge across tasks to escape local optima and boost search efficiency. The main contributions of this work are summarized as follows:

1. A novel Dual-Task Evolutionary Multitasking Optimization (DMLC-MTO) framework is proposed. It balances global exploration and local exploitation to address redundant features and improve search efficiency in high-dimensional spaces.
2. A dynamic multi-indicator evaluation strategy is introduced for auxiliary task construction. It combines Relief-F and Fisher Score with adaptive thresholding to resolve indicator conflicts and select informative features.
3. A hierarchical elite-driven competitive optimization mechanism is designed. It enables intra- and inter-task knowledge transfer to enhance convergence stability and solution quality.

The remainder of this paper is organized as follows. Section 2 reviews related work on high-dimensional feature selection and multi-task optimization. Section 3 introduces the proposed dynamic multitask learning framework, including the task generation strategy, competitive particle swarm optimization with hierarchical elite learning, and the knowledge transfer mechanism. Section 4 presents experimental results and analysis on benchmark datasets. Finally, Section 5 concludes the paper and discusses future research directions.

## 2 Related work

### 2.1 Problem formulation

Feature selection aims to identify a subset of informative features from a high-dimensional feature space while removing redundant or irrelevant ones. Formally, given a dataset $D\in \{(x_{i},y_{i})\}$ , i=1,⋯,n , where $x_{i}\in {\mathbb{R}}^{d}$ is a feature vector and $y_{i}$ is the corresponding label, the objective is to find a binary selection vector $z\in {\left\{0,1\right\}}^{d}$ such that the selected subset $S=\{j|z_{j}=1\}$ maximizes model performance with minimal feature count. This problem is inherently combinatorial and becomes more challenging as dimensionality increases, especially when feature relevance is sparse or context-dependent.

Due to the exponential number of possible feature subsets, feature selection is considered an NP-hard problem. In high-dimensional scenarios, especially when the number of features greatly exceeds the number of samples, the presence of redundant, noisy, or irrelevant features can severely degrade model performance and increase computational cost. Therefore, effective feature selection is critical for improving model generalization, enhancing interpretability, and reducing overfitting risks in complex learning tasks.

### 2.2 Related work

Over the past decades, numerous FS algorithms have been developed, which can be broadly categorized into filter, wrapper, and evolutionary-based methods.

Filter methods select features based on their intrinsic properties such as correlation, information entropy, or discriminative power, independent of any classifier. Common approaches include correlation-based feature selection (CFS) (Hall, 1999), mutual information (Vergara and Estévez, 2014), and Gini Index (Solorio et al., 2020). Relief-F (Kononenko, 1994), one of the earliest and most influential methods, ranks features by assessing how well they distinguish between instances of different classes. It demonstrates strong robustness to noise and applicability across different learning models. To alleviate feature redundancy, CFS (Hall, 1999) evaluates feature subsets by considering both individual relevance and pairwise correlations. The fast correlation-based filter (FCBF) (Senliol et al., 2008) further improves efficiency by rapidly removing redundant features based on entropy-based measures. In more complex scenarios involving mixed-type data, hybrid methods such as SFSDFC (Yan et al., 2021) and UFS (Solorio et al., 2024) have been proposed to integrate density-based clustering and spectral analysis for robust feature evaluation. Nevertheless, filter methods often suffer from suboptimal feature subset selection due to the lack of interaction with model performance.

Wrapper methods evaluate subsets of features using specific learning algorithms, offering better performance in classification tasks. Sequential forward selection (Guan et al., 2004), sequential backward selection (SBS) (Fernández-Diego and González-Ladrón-de-Guevara, 2018), and recursive feature elimination (RFE) (Paul and Dupont, 2015) are classical examples. RFE, in particular, uses classifiers such as support vector machines to recursively remove the least important features, delivering high accuracy but with high computational cost. Hybrid and heuristic wrappers have emerged to improve efficiency, including mixed forward selection (MFS) (Tang and Mao, 2007), binomial cuckoo search (Pandey et al., 2020), and binary Jaya with TOPSIS decision logic (Chaudhuri and Sahu, 2021). Metaheuristic-based methods, such as Firefly Algorithm and Hyena Optimization (Lohitha and Pounambal, 2022), have also been used to balance search quality and complexity. Although wrapper methods provide better feature subsets, they often become infeasible on large-scale or high-dimensional data due to their high time complexity.

Evolutionary algorithm such as PSO and CSO have been widely adopted for feature selection due to their capability in handling large search spaces (Huda and Banka, 2019; Tran et al., 2019; Swesi and Bakar, 2019; Song et al., 2021; Too et al., 2019). PSO variants have been enhanced with rough sets (Huda and Banka, 2019), adaptive subpopulation strategies (Tran et al., 2019), and feature clustering (Swesi and Bakar, 2019). Meanwhile, CSO has been improved with binary encoding (Too et al., 2019), genetic operators (Ding et al., 2020), and chaotic functions (Pichai et al., 2020) to increase diversity and convergence speed. In addition, PSO and other metaheuristic-based algorithms have also shown strong adaptability in broader application domains, such as cloud resource forecasting (Salb et al., 2024), software defect prediction (Villoth et al., 2025), sentiment classification (Mladenovic et al., 2024), and intrusion detection in IoT systems (Dakic et al., 2024).

However, these methods still suffer from premature convergence and inefficient exploration in ultra-high-dimensional settings. As a response, evolutionary multitasking (EMT) strategies have been introduced to feature selection problems. Chen proposed multitask PSO methods (Chen et al., 2020; Chen et al., 2021) that convert high-dimensional feature selection into correlated subtasks and facilitate knowledge transfer between them. More recently, Li extended this idea by integrating filter-based indicators to generate diverse auxiliary tasks (Li et al., 2023), further improving optimization performance on high-dimensional datasets. However, most existing MTL or transfer-based FS methods still rely on fixed task definitions and lack adaptive mechanisms for dynamic task construction, relevance evaluation, and selective transfer.

In addition, few works consider integrating multiple evaluation criteria to construct feature relevance measures dynamically. The conflict between different indicators (e.g., Relief-F vs. Fisher Score) often leads to inconsistent selection results. Furthermore, evolutionary optimization in multitask FS scenarios still faces challenges such as inefficient exploration, insufficient exploitation of inter-task knowledge, and the risk of negative transfer.

Despite the progress made, several challenges in high-dimensional feature selection remain insufficiently addressed. These include how to construct auxiliary tasks in a data-driven manner using multiple relevance indicators, how to facilitate effective yet selective knowledge transfer between tasks, and how to improve search efficiency without compromising solution quality. This work explores these aspects by proposing a multitask optimization framework that incorporates multi-criteria based task construction, competitive learning with hierarchical elites and elite-based knowledge transfer strategy to enhance the feature selection process.

## 3 The proposed algorithm

### 3.1 Main framework of the proposed algorithm

To address the challenges of high-dimensional feature selection, we propose a novel evolutionary multitasking optimization framework, DMLC-MTO (dynamic multitask learning via competitive elites) is proposed. The core idea is to jointly explore the global feature space and exploit locally informative subsets through a dual-task structure, enhanced by elite-guided search.

**Figure 1:**
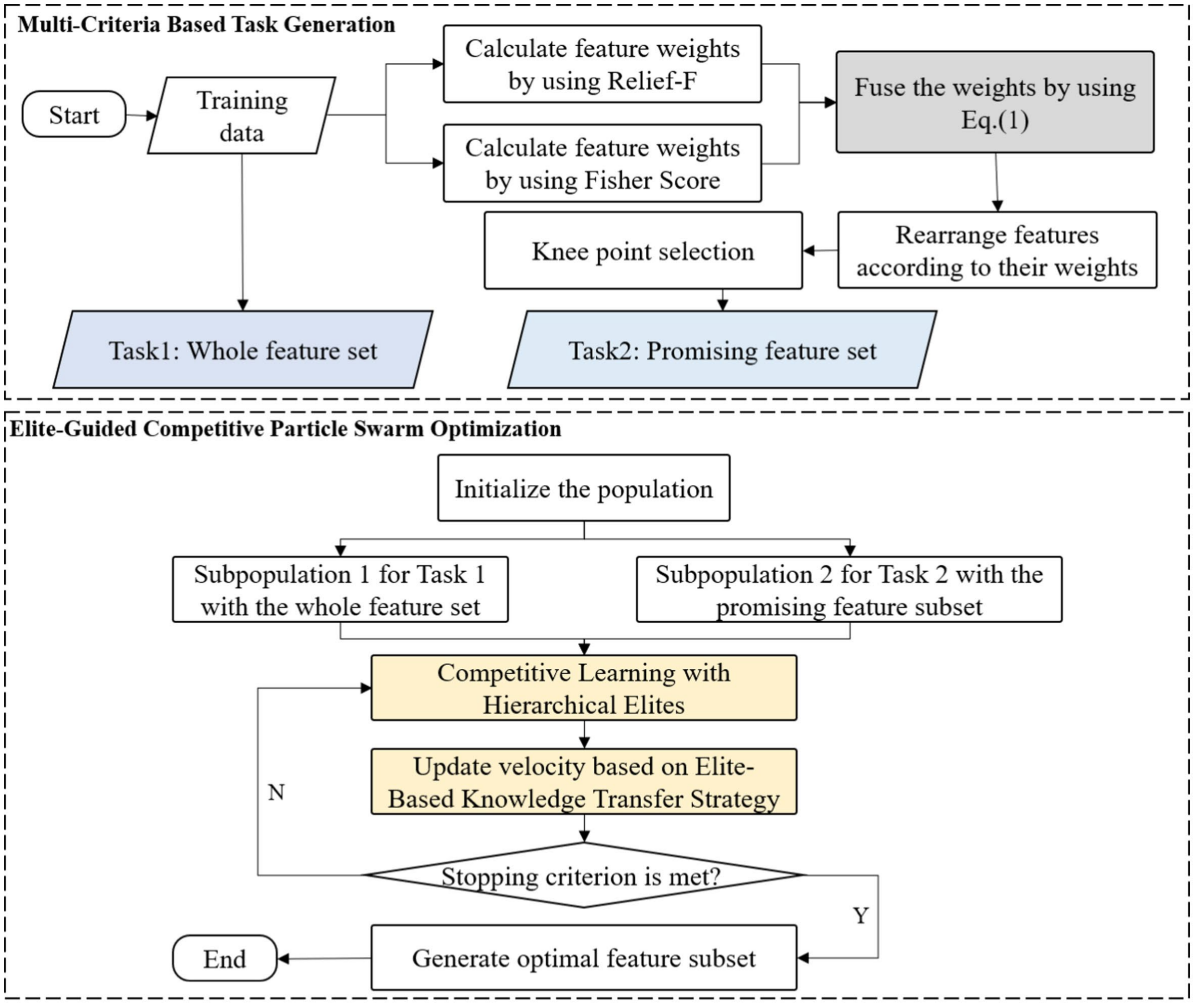
The framework of the proposed DMLC-MTO.

As illustrated in Figure 1, DMLC-MTO operates on two complementary tasks. The primary task performs global feature optimization in the full feature space, while the auxiliary task focuses on a reduced subspace constructed via a Multi-Criteria based Task Generation Strategy. This task division allows the algorithm to simultaneously capture broad feature relevance and fine-grained local discriminability.

Both tasks evolve independently through competitive particle swarm optimization but interact dynamically via an elite-based knowledge sharing mechanism. High-quality solutions from one task can influence the search direction of the other, enabling mutual reinforcement and improving convergence behavior. The overall workflow of DMLC-MTO is outlined in Algorithm 1.

**ALGORITHM 1:**
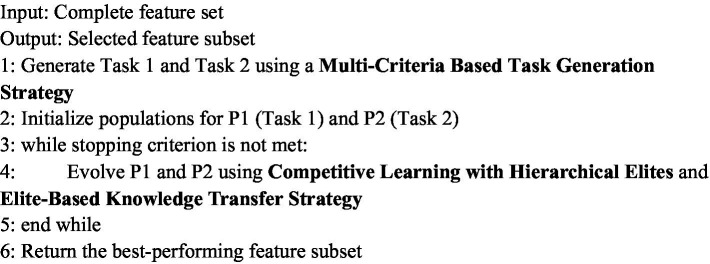
DMLC-MTO optimization framework.

### 3.2 Multi-criteria based task generation strategy

In high-dimensional feature selection, designing an effective auxiliary task is a key challenge for multitask optimization. Naively increasing the number of tasks often leads to unnecessary computational overhead and increases the risk of negative transfer. To address this, DMLC-MTO adopts a two-task paradigm: the primary task operates on the full feature space to ensure global exploration, while the auxiliary task focuses on a compact subset of features to enable refined local optimization. A critical factor in the success of this paradigm is how the auxiliary feature subset is constructed.

Traditional evaluation methods, such as Relief-F and Fisher Score, offer different perspectives on feature importance. Relief-F emphasizes neighborhood-based instance discrimination, while Fisher Score captures between-class variance. However, these approaches often produce inconsistent feature rankings when used independently, particularly in high-dimensional settings where feature redundancy and noise are common. To mitigate these inconsistencies, this work proposes a Multi-Criteria Based Task Generation Strategy that integrates both metrics into a unified scoring framework.

As shown in Equation 1 the strategy begins by independently computing the importance weights of each feature using both Relief-F and Fisher Score. These two score vectors are then fused using a weighted linear combination.


(1) $$w_{i}=\alpha \cdot w_{i}^{\mathrm{RF}}+\beta \cdot w_{i}^{\mathrm{FS}}$$

Where $w_{i}\;$ denotes the combined weight of the *i*-th feature, *α* and *β* control the relative contribution of each metric, allowing flexible adjustment based on data characteristics. This yields a comprehensive feature relevance score for each feature.

**Figure 2:**
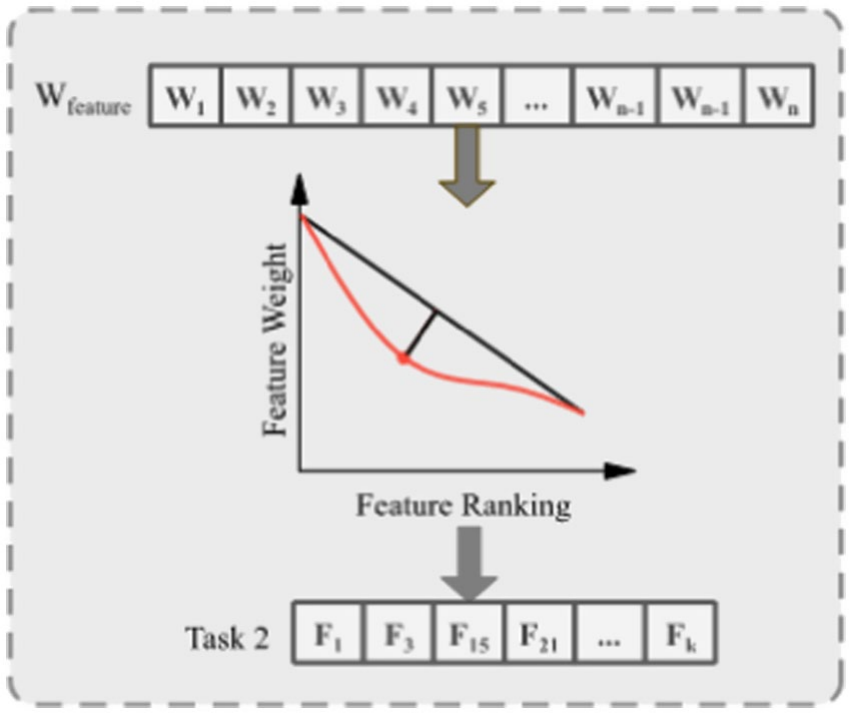
Feature score curve and knee point detection for adaptive thresholding.

Instead of arbitrarily selecting a fixed number of features (e.g., top-k), the proposed strategy employs knee point detection to adaptively determine the weight threshold for feature selection, ensuring both focus and relevance. As illustrated in Figure 2, each feature’s score is first calculated and ranked in descending order. A curve is plotted based on these scores, and a straight line is drawn connecting the highest and lowest points. The feature corresponding to the maximum perpendicular distance between the curve and the line, which is the knee point and marked as the red point in Figure 2, is identified as the selection threshold. Features with scores above this threshold are considered the most significant and are subsequently selected for Task 2.

This adaptive thresholding technique ensures that the selected features are statistically meaningful and tailored to the data distribution, avoiding arbitrary cutoffs and improving robustness. As a result, Task 2 is constructed using only the features above the knee point, while Task 1 retains the full feature set. This design ensures that the auxiliary task remains focused and efficient, while the primary task provides broader coverage, allowing the multitask optimization process to benefit from both global and fine-grained representations.

### 3.3 Elite-guided competitive particle swarm optimization

High-dimensional feature selection presents two key challenges for evolutionary algorithms: premature convergence and inefficient exploration. Standard PSO often struggles in such settings, especially when the feature space is sparse or the objective landscape is complex and multimodal. To overcome these limitations, the proposed DMLC-MTO framework incorporates an enhanced optimization strategy called elite-guided competitive PSO (EC-PSO), which integrates competitive learning with hierarchical elites and cross-task knowledge transfer.

#### 3.3.1 Competitive learning with hierarchical elites

**Figure 3:**
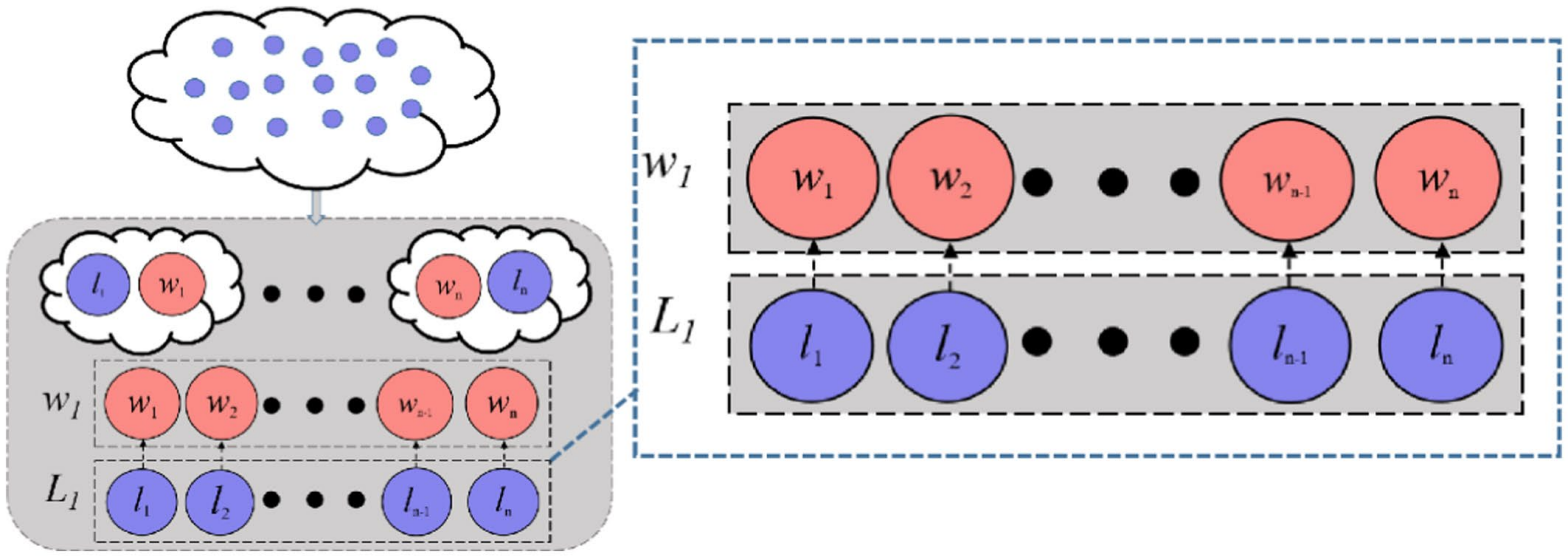
Competition among particles in classical CSO.

The EC-PSO builds upon the CSO, which introduces pairwise competitions among particles. As shown in Figure 3, in each generation, particles are randomly grouped into pairs. Within each pair, the particle with superior fitness is marked as the winner, and the other as the loser. The loser updates its velocity and position by learning from the winner and the population centroid, according to Equations 2, 3:


(2) $$\begin{array}{l} V_{L}(t+1)=r_{1}\times V_{L}(t)+r_{2}\times (X_{W}(t)-X_{L}(t))\\ \;+\varphi \times r_{3}\times (\bar{X}(t)-X_{L}(t)) \end{array}$$

(3) $$X_{L}(t+1)=X_{L}(t)+V_{L}(t+1)$$

where *r*_1_,*r*_2_,*r*_3_∈[0,1] are random coefficients, $X_{W}(t)$ is the winner’s position, $\bar{X}(t)$ is the average position of all particles, and $V_{L}(t)$ , $X_{L}(t)$ denote the velocity and position of the loser, respectively, φ controls the influence of $\bar{X}(t)$ . This formulation ensures convergence toward both local optima (through direct winner imitation) and the population mean (to maintain diversity).

**Figure 4:**
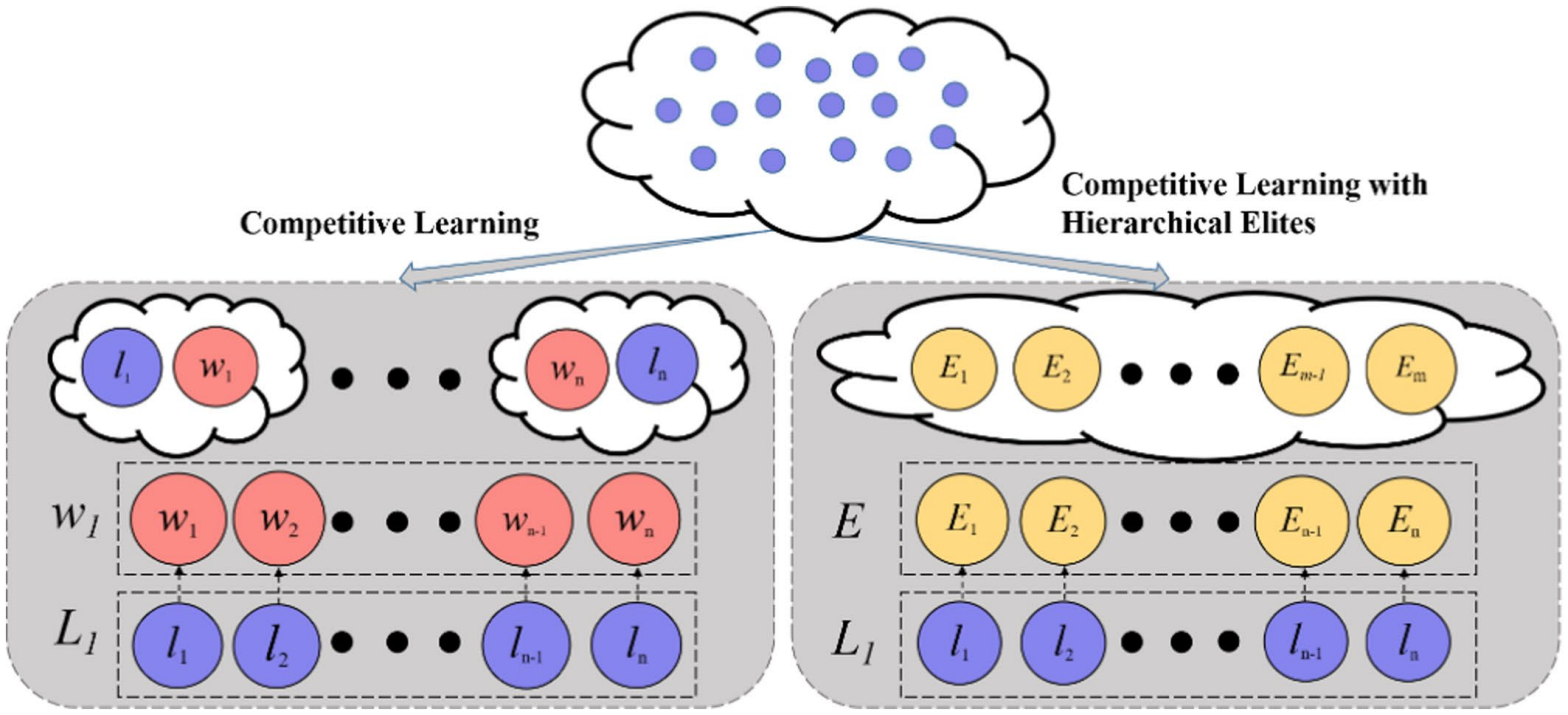
Competitive learning with hierarchical elites.

However, standard CSO lacks global guidance and can stagnate when the winner’s solution quality plateaus. To enhance the convergence performance in high-dimensional feature selection, DMLC-MTO incorporates an elite-driven competitive learning mechanism. As shown in Figure 4, in each optimization iteration, the algorithm identifies the top-K best-performing particles in each task to form a task-specific elite pool. After applying the pairwise competition strategy, each loser particle is given a probability 𝑃𝑘 to learn from a randomly selected particle in the elite pool instead of the winner in its own pair. This elite-level guidance introduces a long-term memory mechanism that directs losers toward globally promising solutions, helping the swarm avoid local stagnation.

#### 3.3.2 Elite-based knowledge transfer strategy

In multitask optimization, knowledge transfer between tasks plays a vital role in improving overall search performance by sharing successful patterns. To this end, DMLC-MTO introduces a hierarchical elite-based transfer strategy. Specifically, for each loser particle, a random number is generated and compared with a predefined transfer probability 𝑃trans. If the transfer condition is satisfied, the particle is allowed to learn from elite solutions of another task, rather than only relying on intra-task updates.

Furthermore, within both intra-task and cross-task settings, the algorithm checks whether another random value exceeds the elite learning threshold 𝑃𝑘. If so, the particle learns from an elite particle; otherwise, it learns from the average position of all particles or elites in the corresponding task. The update strategy thus includes four distinct modes as Equations 4–7:


(4) $$\begin{array}{c} V_{L}^{i}(t+1)=r_{1}\times V_{L}(t)+r_{2}\times (X_{\text{Erand}}^{\text{task}}(t)-X_{L}^{i}(t))\\ +r_{3}\times (X_{\text{Erand}}(t)-X_{L}(t)) \end{array}$$

(5) $$\begin{array}{c} V_{L}^{i}(t+1)=r_{1}\times V_{L}(t)+r_{2}\times (X_{W}^{\text{task}}(t)-X_{L}^{i}(t))\\ +r_{3}\times (X_{\text{wrand}}(t)-X_{L}(t)) \end{array}$$

(6) $$\begin{array}{c} \begin{array}{c} V_{L}^{i}(t+1)=r_{1}\times V_{L}(t)+r_{2}\times (X_{\text{Erand}}^{\text{task}}(t)-X_{L}^{i}(t))\\ +\varphi \times r_{3}\times ({\bar{X}}_{E}^{\text{task}}(t)-X_{L}(t)) \end{array} \end{array}$$

(7) $$\begin{array}{c} V_{L}^{i}(t+1)=r_{1}\times V_{L}(t)+r_{2}\times (X_{W}^{\text{task}}(t)-X_{L}^{i}(t))\\ +\varphi \times r_{3}\times (\bar{X}(t)-X_{L}(t)) \end{array}$$

Where $X_{\text{Erand}}^{\text{task}}(t)$ a particle randomly selected from the elite pool within the current task, while $X_{\text{Erand}}(t)$ is randomly chosen from the elite pool of other tasks, $X_{W}^{\text{task}}(t)$ denotes the winner particle within the same task, $X_{\text{wrand}}(t)$ is a randomly selected winner from other tasks, represents the average position of elites in the current task, $\bar{X}(t)$ denotes the average position of all particles in the current population, φ serves as a control factor to regulate the influence of the corresponding learning component. From Equations 4–7, it can be observed that each particle updates its position by simultaneously learning from two types of sources. By incorporating knowledge from the winner in the same task, the winner from another task, the average position of elite particles within the current task, and the overall population mean, the particle significantly enhances its search capability. This design helps maintain population diversity and improves optimization efficiency.

### 3.4 Fitness function

An effective fitness function plays a vital role in guiding the evolutionary search toward an optimal feature subset. In this study, we adopt an evaluation strategy that considers both classification performance and feature compactness is adopted. The overall fitness function is defined as Equations 8, 9:


(8) $$\text{fitness}=\alpha_{f}*\mathrm{\gamma R}(D)+(1-\alpha_{f})*\frac{|S|}{|N|}$$

(9) $$\mathrm{\gamma R}(D)=1-\frac{1}{C}*\sum_{i=1}^{C}{\mathrm{TPR}}_{i}$$

Where γR(D) denotes the probability of classification error. The term ∣S∣ indicates the number of selected features used to construct the model, while ∣N∣ corresponds to the total number of available features in the dataset. The parameter $\alpha_{f}$ , ranging between 0 and 1, controls the trade-off between classification accuracy and feature sparsity. Following the recommendation in Chen et al. (2019), *α* is set as 0.999999 to place a stronger emphasis on classification performance. The number of classes C reflects the total distinct categories involved in the classification task. For each class *i*, the true positive rate ${\mathrm{TPR}}_{i}$ is computed as the proportion of correctly predicted instances within that class relative to the total number of samples in the same class (Patterson and Zhang, 2007). Using the balanced error metric mitigates the influence of class imbalance, ensuring that all classes contribute equally to the evaluation. This is particularly important in feature selection, where biased evaluation can lead to overfitting to majority classes and suboptimal feature subsets.

## 4 Experimental results and analysis

### 4.1 Experimental setup

To comprehensively evaluate the performance of the proposed algorithm, experiments were conducted on 13 high-dimensional real-world datasets drawn from various application domains. These datasets exhibit a wide range of feature dimensions, varying from 2,000 to over 13,000, making them suitable for assessing the algorithm’s effectiveness under diverse and complex conditions. Detailed characteristics of each dataset—including the dataset name, number of features, number of samples, and number of classes—are summarized in Table 1.

**Table 1:** Dataset.

No.	Dataset	Feature no.	Instance no.	Class no.
1	SRBCT	2,308	83	4
2	warpPIE10P	2,420	210	10
3	Lymphoma	5,026	62	3
4	Nci	5,244	61	8
5	Leukemia 1	5,327	72	3
6	DLBCL	5,469	77	2
7	Prostate6033	6,033	102	2
8	ALLAML	7,129	72	2
9	Nci9	9,712	60	9
10	Orlraws10P	10,304	100	10
11	Prostate	10,509	102	2
12	Leukemia 2	11,225	72	3
13	Lung cancer	12,600	203	5

To evaluate the effectiveness of the proposed DMLC-MTO algorithm, comprehensive comparisons were conducted with four competitive evolutionary algorithm EA-based feature selection methods: PSO (Ansari et al., 2019), CSO-FS (Tian et al., 2019), PSO-EMT (Chen et al., 2020), and MT-PSO (Chen et al., 2021). PSO serves as a baseline representing standard EA strategies, while CSO-FS incorporates the traditional CSO search mechanism specifically designed for feature selection. PSO-EMT and MT-PSO both adopt multitask learning paradigms to better address the challenges of high-dimensional feature spaces, and have demonstrated competitive performance in previous studies. All experiments were implemented in MATLAB R2020a and executed on a Windows 10 machine with a 2.6 GHz Intel Core i5 processor and 16GB RAM, ensuring a consistent computational environment across all methods. To prevent feature selection bias and ensure an unbiased evaluation, feature selection was performed separately within each training fold during cross-validation. Specifically, for each fold, the training data was used to generate tasks and perform feature selection, and the resulting selected features were then applied to the held-out test fold for performance evaluation. This nested-like procedure ensures that no information from the test set is used during feature selection, thereby providing a reliable estimate of the algorithm’s generalization performance.

In addition to these methods, this work included a baseline classifier without any feature selection (referred to as FULL) to highlight the improvements achieved by each FS approach in terms of classification accuracy and dimensionality reduction. All experimental results were obtained from 30 independent runs to account for algorithmic stochasticity. For statistical validation, the Wilcoxon signed-rank test was applied at a significance level of 0.05. In the analysis, the symbols “+,” “−,” and “=” denote that a comparison method performs significantly better, worse, or comparable to the proposed DMLC-MTO, respectively.

To ensure fair and consistent comparisons, all algorithms were evaluated under standardized experimental settings. Each task was executed with a population size of 70 and a maximum of 100 iterations. Additionally, the proposed algorithm is model-agnostic and compatible with various classifiers, making it adaptable to different application scenarios without restricting the choice of classification models. The complete parameter configurations for all methods are summarized in Table 2 to support reproducibility and facilitate future implementation.

**Table 2:** Algorithms parameters setting.

Algorithms	Parameters
PSO	$c_{1}=c_{2}=c_{3}=1.49445$ $w=0.9-0.5\times (\text{iter}/n_{\text{iter}})$
CSO-FS	$r_{1},r_{2},r_{3}\in [0,1]$
PSO-EMT	$c_{1}=c_{2}=c_{3}=1.49445,\rho =0.05,\mathrm{rmp}=0.6,m=10$ $w=0.9-0.5\times (\text{iter}/n_{\text{iter}})$
MT-PSO	$c_{1}=c_{2}=c_{3}=1.49445,\rho =0.05,\mathrm{rmp}=0.6,G=6$ $w=0.9-0.5\times (\text{iter}/n_{\text{iter}})$
DMLC-MTO	$r_{1},r_{2},r_{3}\in [0,1]$ P=0.6 *Pk* = 0.6 $\alpha_{f}=0.999999$

### 4.2 Comparison with the state-of-the-art algorithms

#### 4.2.1 Classification accuracy comparison

Table 3 presents the classification accuracy of DMLC-MTO compared to four baseline algorithms across multiple datasets. Among 65 comparisons, DMLC-MTO outperforms the other methods in 56 cases, achieves comparable results in 8, and underperforms in only 1 case. These results demonstrate the overall superiority and robustness of the proposed method. A detailed analysis is as follows:

**Table 3:** Classification accuracy of the compared algorithms on multiple datasets.

Dataset	FULL	PSO	CSO-FS	PSO-EMT	MT-PSO	DMLC-MTO
SRBCT	80 (−)	95.27 (=)	95.13 (=)	95.12 (=)	95.21 (=)	**95.3**
warpPIE10P	83.58 (−)	98.17 (−)	51.54 (−)	99.12 (=)	99.21 (=)	**99.13**
Lymphoma	99.08 (−)	81.35 (−)	55.14 (−)	93.26 (−)	96.51 (−)	**99.22**
Nci	66.46 (−)	65.36 (−)	64.12 (−)	59.32 (−)	63.87 (−)	**68.98**
Leukemia 1	78.74 (−)	80.47 (−)	81.27 (−)	86.08 (−)	86.12 (−)	**87.86**
DLBCL	82.79 (−)	83.72 (−)	83.92 (−)	85.17 (−)	87.67 (−)	**89.78**
Prostate6033	82.11 (−)	84.07 (−)	83.68 (−)	80.57 (−)	84.07 (−)	**86.66**
ALLAML	78.06 (−)	79.44 (−)	82.02 (−)	89.9 (−)	91.16 (−)	**94.1**
Nci9	41.3 (−)	47.32 (−)	43.34 (−)	51.11 (=)	51.21 (=)	**51.15**
Orlraws10P	78.32 (−)	90.84 (−)	92.09 (−)	93.37 (−)	93.18 (−)	**98.2**
Prostate	84.34 (−)	82.65 (−)	**88.36 (=)**	81.68 (−)	84.17 (−)	88.38
Leukemia 2	87.68 (−)	87.15 (−)	85.46 (−)	87.16 (−)	86.45 (−)	**88.12**
Lung cancer	77.65 (−)	78.41 (−)	78.47 (−)	83.47 (−)	84.75 (−)	**87.22**
+/−/=	0/13/0	0/12/1	1/11/1	0/10/3	0/10/3	

Best results are highlighted in bold.

Compared with FULL: DMLC-MTO consistently outperforms the baseline classifier without feature selection (FULL) on all 13 datasets, highlighting the necessity and effectiveness of feature selection. For example, on the NCI9 dataset, DMLC-MTO achieves an accuracy of 68.98%, improving upon FULL’s 66.46% by 3.8%. On the high-dimensional Prostate6033 dataset, DMLC-MTO reaches 86.66%, surpassing FULL’s 82.11% by 5.54%. These improvements suggest that assigning discriminative weights to features effectively mitigates the curse of dimensionality and enhances classification performance.

Compared with PSO: DMLC-MTO significantly outperforms standard PSO on 12 out of 13 datasets, with the only tie occurring on the SRBCT dataset (95.3% vs. 95.27%). On the Lymphoma dataset, DMLC-MTO achieves 99.22%, markedly higher than PSO’s 81.35%, a relative improvement of 21.97%. Similarly, it reaches 98.2% on the Orlraws10P dataset, outperforming PSO’s 90.84% by 7.5%.

Compared with CSO-FS: DMLC-MTO exhibits clear advantages over CSO-FS, achieving better performance on 11 out of 16 datasets, with only one dataset where it performs slightly worse and the rest showing comparable results. On average, DMLC-MTO improves classification accuracy by 11.55 percentage points over CSO-FS. This gain underscores the effectiveness of the multitask framework and the embedded elite-driven competition mechanism in improving feature selection.

Compared with PSO-EMT: DMLC-MTO outperforms PSO-EMT on 10 out of 13 datasets, with equivalent performance on NCI9, Prostate6033, and one additional dataset. For example, it achieves 99.22% accuracy on Lymphoma, exceeding PSO-EMT’s 93.26% by 6.4%, and obtains 89.78% on DLBCL, outperforming PSO-EMT’s 85.17% by 5.4%. These improvements are largely attributed to the hierarchical elite learning strategy, which dynamically guides the search towards more informative feature subsets.

Compared with MT-PSO: DMLC-MTO achieves superior accuracy on 10 out of 13 datasets compared to MT-PSO, with tied performance on NCI9, Prostate6033, and one other dataset. On the ALLAML dataset, DMLC-MTO reaches 94.1%, improving upon MT-PSO’s 91.16% by 3.2%. On Lung Cancer, it records 87.22% versus 84.75% by MT-PSO, a relative gain of 2.9%. These results validate the advantage of DMLC-MTO’s multi-indicator-based task generation strategy, which constructs complementary tasks more effectively than MT-PSO’s random task allocation approach.

#### 4.2.2 Analysis of selected feature subsets

Table 4 reports the number of features selected by different algorithms across all datasets. Reducing the number of selected features often leads to simpler models and improved computational efficiency. The experimental results show that DMLC-MTO consistently selects fewer features than other methods. Among 65 comparisons, it outperforms its competitors in 59 cases, ties in 4, and underperforms in only 3, demonstrating its strong capability in identifying compact and informative feature subsets.

**Table 4:** Number of features selected by different algorithms on multiple datasets.

Dataset	FULL	PSO	CSO-FS	PSO-EMT	MT-PSO	DMLC-MTO
SRCBT	2,308	295.25 (−)	150.2 (−)	122.4 (−)	452.6 (−)	**47.18**
warpPIE10P	2,420	1185.5 (−)	**8.5 (+)**	155.67 (−)	288.6 (−)	32.52
Lymphoma	4,026	2155.1 (−)	**3.5 (+)**	12.52 (+)	21.99 (−)	56.39
Nci	5,244	2064.8 (−)	412.8 (−)	316.74 (−)	1203.47 (−)	**282.12**
Leukemia 1	5,327	2953.4 (−)	435.2 (−)	282.5 (−)	842.56 (−)	**230.22**
DLBCL	5,469	2154.7 (−)	332.25 (−)	194.4 (=)	1025.27 (−)	200.22
Prostate6033	6,033	3158.4 (−)	780.54 (−)	462.36 (−)	1451.12 (−)	**113.82**
ALLAML	7,129	3834.1 (−)	392.5 (−)	162.82 (−)	1852.22 (−)	**79.93**
Nci9	9,712	4426.1 (−)	618.4 (+)	1932.7 (=)	613.28 (+)	1947.22
Orlraws10P	10,304	4834.9 (−)	65.4 (=)	932.86 (−)	1208.04 (−)	**63.28**
Prostate	10,509	5675.5 (−)	1305.4 (−)	185.12 (−)	2759.71 (−)	**231.32**
Leukemia 2	11,225	3548.1 (−)	1057.6 (−)	298.3 (=)	1587.2 (−)	**285.58**
Lung cancer	12,600	6923.8 (−)	1168.4 (−)	753.38 (−)	832.4 (−)	**322.48**
+/−/=		0/13/0	2/9/2	1/11/1	0/12/1	

Best results are highlighted in bold.

On high-dimensional datasets such as Orlraws10P and Lung Cancer (with over 10,000 dimensions), DMLC-MTO achieves significant dimensionality reduction while maintaining high classification accuracy. This demonstrates not only its effectiveness in compressing features, but also its ability to filter out redundant or irrelevant attributes and retain the most discriminative ones. Such compact representations are particularly beneficial in practical scenarios where computational resources are limited or real-time decision-making is required. Moreover, reducing the number of features improves model interpretability, which is critical in domains like healthcare and bioinformatics.

Although DMLC-MTO performs well on most datasets, there are a few cases where its classification accuracy is slightly lower than that of CSO-FS. For instance, on the Prostate dataset, DMLC-MTO selects an average of 132.13 features compared to 1305.4 selected by CSO-FS. Despite selecting far fewer features, DMLC-MTO’s accuracy is marginally lower. This may be attributed to its limited ability to capture complex nonlinear dependencies between features when relying on filter-based metrics like Fisher Score. Furthermore, the competitive learning mechanism may occasionally introduce negative transfer, where informative features are prematurely discarded in early stages of optimization, slightly affecting final performance.

Nonetheless, the global search capability of DMLC-MTO ensures that it can still find effective solutions with fewer features. On datasets such as warpPIE10P and Lymphoma, where CSO-FS selects fewer features (8.5 and 3.5, respectively), it achieves only 51.54% and 55.14% accuracy. In contrast, DMLC-MTO selects slightly more features (34.57 and 86.74, respectively), yet reaches significantly higher accuracies of 99.56% and 99.36%. These results highlight the strength of DMLC-MTO’s dual-task and elite-guided learning mechanisms in discovering high-quality feature combinations that lead to better overall performance.

In summary, DMLC-MTO shows great potential for high-dimensional feature selection, especially in tasks that require a minimal number of features without sacrificing classification performance. By significantly reducing dimensionality while maintaining or improving predictive accuracy, DMLC-MTO demonstrates an effective trade-off between model simplicity and discriminative power. These findings further confirm the algorithm’s suitability for practical applications and provide a solid foundation for future research.

#### 4.2.3 Training time comparison

**Figure 5:**
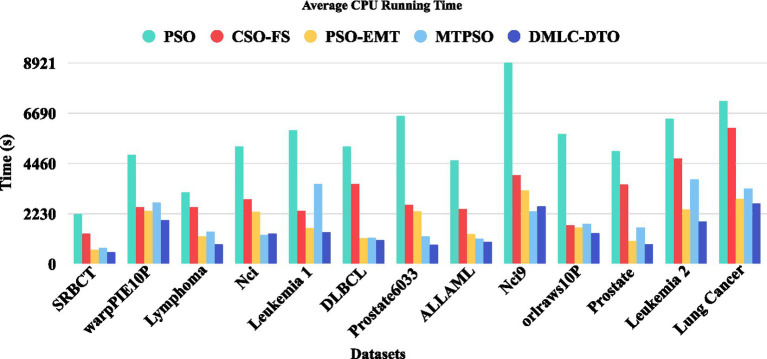
Average CPU running time.

Figure 5 presents the training time comparison among all algorithms, highlighting the computational efficiency of DMLC-MTO. Benefiting from its integrated multitask mechanism and efficient search dynamics, DMLC-MTO significantly reduces redundant computations while maintaining solution quality, leading to superior runtime performance across all datasets.

### 4.3 Mechanism analysis of DMLC-DTO

#### 4.3.1 Evaluation of multi-criteria task generation strategy

To assess the effectiveness of the proposed multi-criteria task generation strategy, it is compared against three widely used feature evaluation methods: Relief-F, Pearson Correlation Coefficient (PCC), and Total Variance (TV). The comparison was conducted across 13 datasets of varying dimensionality, evaluating both the size of the selected feature subset and the resulting classification accuracy. The detailed results are presented in Table 5.

**Table 5:** Comparison of the different task generation methods.

Dataset	Method	Subset no.	Accuracy
SRBCT	Relief-F	54.4	95.23
	PCC	70.5	94.58
	TV	87.1	88.33
	Multi-criteria task generation	**47.18**	**95.3**
warpPIE10P	Relief-F	43.7	98.5
	PCC	42.3	98.2
	TV	70.8	97.3
	Multi-criteria task generation	**32.52**	**99.13**
Lymphoma	Relief-F	97.2	99.16
	PCC	83.2	98.9
	TV	81.6	98.5
	Multi-criteria task generation	**56.39**	**99.33**
Nci	Relief-F	**133.2**	60.57
	PCC	160.8	**70.04**
	TV	184	66.02
	Multi-criteria task generation	282.12	68.98
Leukemia 1	Relief-F	122.7	87.77
	PCC	156.8	88.35
	TV	**120.6**	88.11
	Multi-criteria task generation	230.22	**87.86**
DLBCL	Relief-F	307.8	86.5
	PCC	179.2	**89.83**
	TV	524.4	85.33
	Multi-criteria task generation	**158.33**	89.56
Prostate6033	Relief-F	191.2	86.16
	PCC	133.5	86.33
	TV	251.5	85.5
	Multi-criteria task generation	**113.82**	**86.66**
ALLAML	Relief-F	95.4	88.41
	PCC	111.8	93.16
	TV	152.7	91.16
	Multi-criteria task generation	**79.93**	**94.10**
Nci9	Relief-F	1438.2	46.22
	PCC	1194.1	51.14
	TV	**1074.4**	**52.09**
	Multi-criteria task generation	1947.22	51.12
Orlraws10P	Relief-F	136.9	94.2
	PCC	305.5	92.5
	TV	100.8	96.1
	Multi-criteria task generation	**63.28**	**98.2**
Prostate	Relief-F	**174.8**	82.16
	PCC	185.3	84.5
	TV	524.4	85.33
	Multi-criteria task generation	231.32	**88.38**
Leukemia 2	Relief-F	354.9	**91.66**
	PCC	387	88.33
	TV	298.3	88.33
	Multi-criteria task generation	**285.58**	88.12
Lung cancer	Relief-F	222.7	79.13
	PCC	434.4	86.08
	TV	**126.9**	82.71
	Multi-criteria task generation	322.48	**87.22**

Best results are highlighted in bold.

Overall, the proposed strategy demonstrates competitive or superior performance across most datasets. In terms of classification accuracy, the multi-criteria approach achieves the highest score in seven out of 13 datasets, and performs comparably in five others. For instance, on the warpPIE10P dataset, it achieves 99.13% accuracy using only 32.52 features, outperforming all baseline methods in both accuracy and feature compactness. Similarly, on Lymphoma, it reaches 99.33% accuracy while reducing the feature subset size to 56.39, significantly lower than Relief-F’s 97.2 features.

In high-dimensional datasets such as Orlraws10P and DLBCL, the multi-criteria strategy also shows clear advantages. On Orlraws10P, it achieves the highest accuracy (98.2%) with the smallest feature subset (63.28), illustrating the effectiveness of combining multiple relevance indicators to filter redundant features. In DLBCL, it selects fewer features (158.33) than most methods while maintaining high accuracy (89.56%), nearly matching PCC’s best performance (89.83%) with improved compactness.

It is worth noting that in some datasets like Nci9 and Leukemia 2, the proposed method selects a relatively larger number of features. In Nci9, although the feature count increases to 1947.22, the classification accuracy (51.12%) remains competitive with PCC (51.14%) and higher than Relief-F (46.22%). This suggests that, in certain cases, the adaptive thresholding mechanism may favor retaining more features to ensure sufficient representation, especially when the informative features are not well distinguished by individual metrics alone.

Another observation lies in the Prostate6033 and Prostate datasets, where the proposed method outperforms baseline methods in accuracy (e.g., 86.66% and 88.38%, respectively) while also maintaining relatively small feature subsets compared to TV or PCC. This highlights its capacity to balance global relevance and local refinement through dynamic integration of multiple scoring criteria.

In conclusion, the multi-criteria task generation strategy shows strong robustness and adaptability across various datasets. It consistently strikes a favorable trade-off between feature subset size and classification performance. By leveraging the complementary strengths of Relief-F and Fisher Score, and incorporating adaptive knee-point detection, the proposed approach enhances the reliability of feature relevance estimation and improves the initialization of auxiliary tasks in the multitask framework. These results demonstrate that the proposed task construction mechanism is an effective foundation for the DMLC-MTO algorithm.

#### 4.3.2 Elite-guided competitive particle swarm optimization

**Figure 6:**
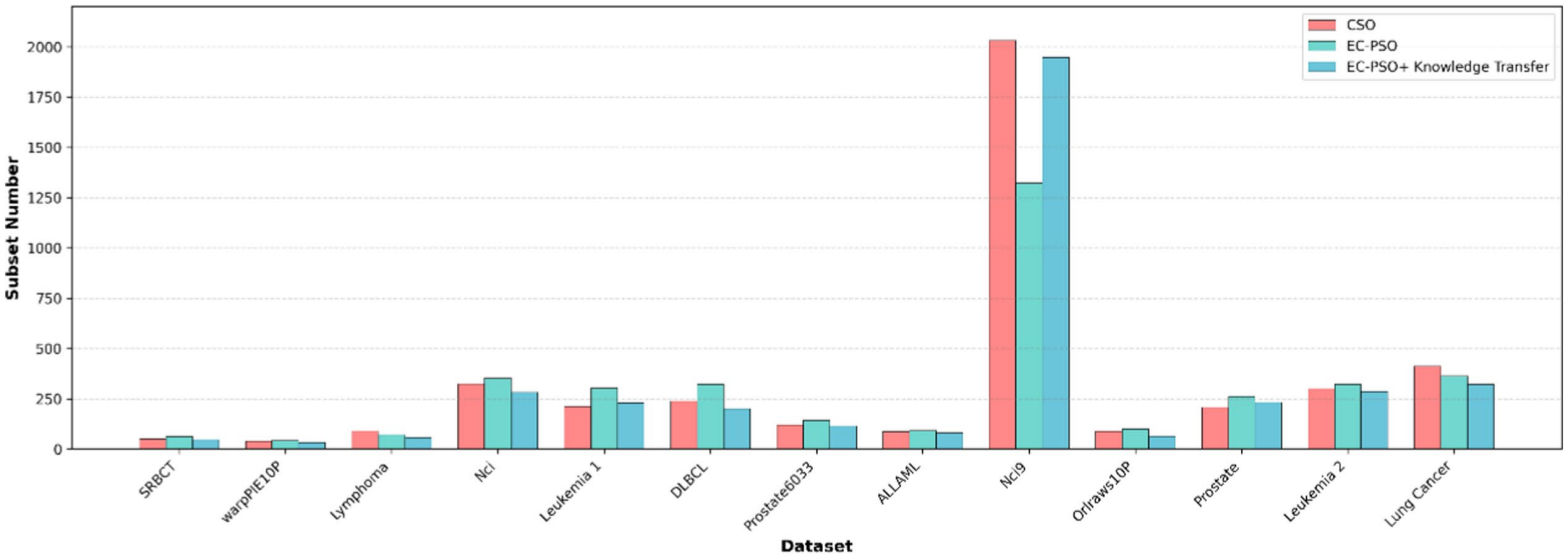
Comparison of the subset number obtained by different optimization and knowledge transfer strategies.

**Figure 7:**
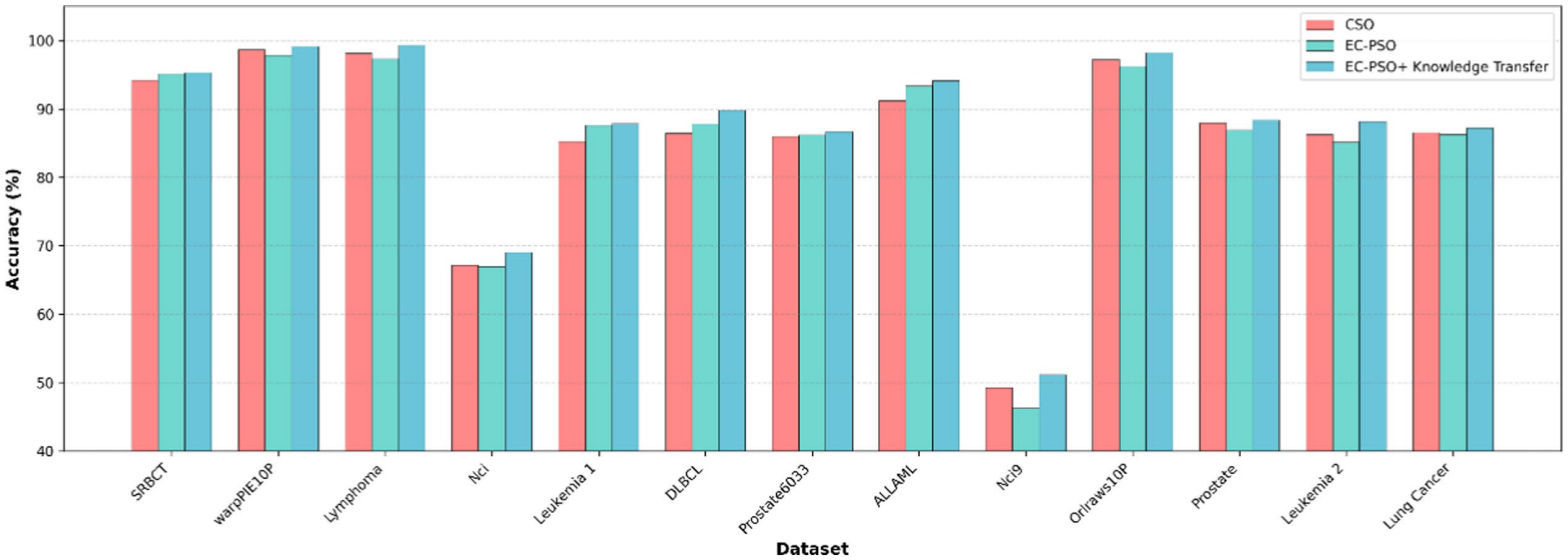
Comparison of accuracy obtained by different optimization and knowledge transfer strategies.

To further evaluate the effectiveness of the proposed knowledge transfer strategy, three variants of the algorithm is considered for comparison: (1) CSO, which relies solely on traditional pairwise competition without elite or task-level interaction; (2) EC-PSO, which introduces elite-based competition but performs optimization independently within each task; and (3) EC-PSO + knowledge transfer, which enhances EC-PSO with inter-task knowledge exchange via elite guidance. The corresponding results are visualized in Figures 6, 7, where the bar charts provide an intuitive comparison of classification accuracy and the number of selected features across different methods. As shown in Table 6, the inclusion of knowledge transfer consistently improves both classification accuracy and feature compactness across most datasets.

**Table 6:** Comparison of the different optimization and knowledge transfer strategies.

Dataset	Method	Subset no.	Accuracy
SRBCT	CSO	50.8	94.16
	EC-PSO	62.32	95.11
	EC-PSO + knowledge transfer	**47.18**	**95.3**
warpPIE10P	CSO	37.86	98.63
	EC-PSO	42.31	97.82
	EC-PSO + knowledge transfer	**32.52**	**99.13**
Lymphoma	CSO	89.42	98.11
	EC-PSO	72.13	97.32
	EC-PSO + knowledge transfer	**56.39**	**99.33**
Nci	CSO	323.86	67.13
	EC-PSO	352.12	66.92
	EC-PSO + knowledge transfer	**282.12**	**68.98**
Leukemia 1	CSO	**212.25**	85.23
	EC-PSO	301.63	87.6
	EC-PSO + knowledge transfer	230.22	**87.86**
DLBCL	CSO	237.36	86.46
	EC-PSO	322.12	87.81
	EC-PSO + knowledge transfer	**200.22**	**89.78**
Prostate6033	CSO	118.76	85.93
	EC-PSO	142.12	86.2
	EC-PSO + knowledge transfer	**113.82**	**86.66**
ALLAML	CSO	86.43	91.22
	EC-PSO	92.11	93.32
	EC-PSO + knowledge transfer	**79.93**	**94.10**
Nci9	CSO	2032.12	49.24
	EC-PSO	**1323.11**	46.23
	EC-PSO + knowledge transfer	1947.22	**51.12**
Orlraws10P	CSO	87.2	97.2
	EC-PSO	99.2	96.2
	EC-PSO + knowledge transfer	**63.28**	**98.2**
Prostate	CSO	**206.4**	87.96
	EC-PSO	261.2	86.91
	EC-PSO + knowledge transfer	231.32	**88.38**
Leukemia 2	CSO	300.32	86.22
	EC-PSO	321.42	85.11
	EC-PSO + knowledge transfer	**285.58**	**88.12**
Lung cancer	CSO	412.12	86.54
	EC-PSO	362.42	86.23
	EC-PSO + knowledge transfer	**322.48**	**87.22**

Best results are highlighted in bold.

While EC-PSO already improves upon CSO by leveraging elite-driven intra-task learning (e.g., on DLBCL and ALLAML datasets), the incorporation of cross-task knowledge sharing in EC-PSO + Knowledge Transfer brings further benefits. It achieves the best performance on nearly all datasets in terms of accuracy, while selecting fewer features in most cases. These results suggest that cross-task learning enables more effective exploration of the search space and promotes better generalization. Therefore, the proposed transfer mechanism not only enhances the performance of individual tasks but also facilitates collaboration between tasks to address the challenges of high-dimensional feature selection.

## 5 Conclusion

This paper presents DMLC-MTO, a dynamic multitask evolutionary feature selection algorithm that integrates multi-indicator task generation, elite competition learning, and spatially-aware knowledge transfer. The framework effectively addresses the challenges of high-dimensional data by enabling precise evaluation of feature subsets and improving optimization efficiency through adaptive inter-task collaboration. Extensive experiments on 13 benchmark datasets demonstrate that DMLC-MTO consistently achieves superior classification accuracy with more compact feature sets compared to existing evolutionary feature selection methods. The combination of complementary filter-based indicators with competitive and transfer mechanisms guides the search toward informative and less redundant features, highlighting the framework’s practical value in applications such as tobacco leaf grading, quality assessment, and agricultural phenotype analysis.

While the results validate the method’s effectiveness, limitations remain. The reliance on filter-based indicators may not fully capture complex nonlinear feature dependencies, some hyperparameters are fixed, and evaluation has been restricted to high-dimensional gene expression and image datasets. Furthermore, qualitative interpretability of the selected features and more rigorous statistical analyses have yet to be explored. Future work will focus on addressing these limitations by incorporating adaptive hyperparameter tuning, evaluating broader and more diverse datasets, enhancing interpretability, and extending the framework to handle multi-label and unsupervised scenarios, thereby strengthening its robustness and broadening its applicability.

## Data Availability

The original contributions presented in the study are included in the article/supplementary material, further inquiries can be directed to the corresponding authors.
